# Idiopathic Intracranial Hypertension Without Papilledema: A Case Emphasizing the Diagnostic Value of Optic Nerve Sheath Ultrasound

**DOI:** 10.7759/cureus.88066

**Published:** 2025-07-16

**Authors:** Asif Mahbub Swapnil, Md Samiul Islam, Latif Rahman, Khalil Fatih Ali Khalil, Nasrul Nijam

**Affiliations:** 1 Acute Medicine, University Hospitals of Leicester National Health Service (NHS) Trust, Leicester, GBR; 2 Medicine, University Hospitals of Leicester National Health Service (NHS) Trust, Leicester, GBR; 3 Acute Medicine, Leicester Royal Infirmary, Leicester, GBR; 4 Neurology, University Hospitals of Leicester National Health Service (NHS) Trust, Leicester, GBR

**Keywords:** cerebrospinal fluid (csf), elevated intracranial pressure, optic nerve sheath, papilloedema, ultrasound imaging

## Abstract

We report the case of a 45-year-old woman with obesity who presented with some features of idiopathic intracranial hypertension (IIH), including postural headache and pulsatile tinnitus, but notably without papilledema. Initial investigations, including optical coherence tomography (OCT) of the optic nerve and computed tomography (CT) of the head, were unremarkable, resulting in a delay in diagnosis. Due to persistent symptoms, optic nerve sheath diameter (ONSD) assessment via ultrasound was performed, revealing bilateral dilation. Subsequent magnetic resonance imaging (MRI) identified an empty sella and distension of the optic nerve sheaths. A lumbar puncture confirmed an elevated opening pressure of 30 cm H₂O, establishing the diagnosis of IIH. This case illustrates the diagnostic challenges of IIH in the absence of papilledema and emphasizes the emerging utility of optic nerve sheath ultrasound as a non-invasive tool to aid in the early detection of raised intracranial pressure.

## Introduction

Idiopathic intracranial hypertension (IIH) is a disorder characterized by raised intracranial pressure (ICP) in the absence of an identifiable cause. It predominantly affects young, obese women, although the exact pathogenesis remains unclear [1]. Clinical symptoms are variable and may include headache, visual disturbances, pulsatile tinnitus, and neck or back pain. Traditionally, diagnosis is based on the presence of papilledema-optic disk swelling caused by raised ICP-typically confirmed by optical coherence tomography (OCT), along with the exclusion of cerebral venous sinus thrombosis (CVST) or other intracranial space-occupying lesions (ICSOLs) on neuroimaging. However, in many resource-limited settings, bedside fundoscopy remains the primary modality for detecting papilledema, despite its lower sensitivity and higher inter-observer variability.

We present the case of a 45-year-old woman with elevated body mass index (BMI) who exhibited symptoms consistent with raised ICP but without papilledema. Her initial diagnostic workup-including OCT-was unremarkable, and IIH was initially ruled out. However, persistent symptoms and further evaluation, including optic nerve sheath diameter (ONSD) ultrasound, revealed features suggestive of raised ICP, ultimately leading to a revised diagnosis of IIH.

## Case presentation

**Figure 1:**
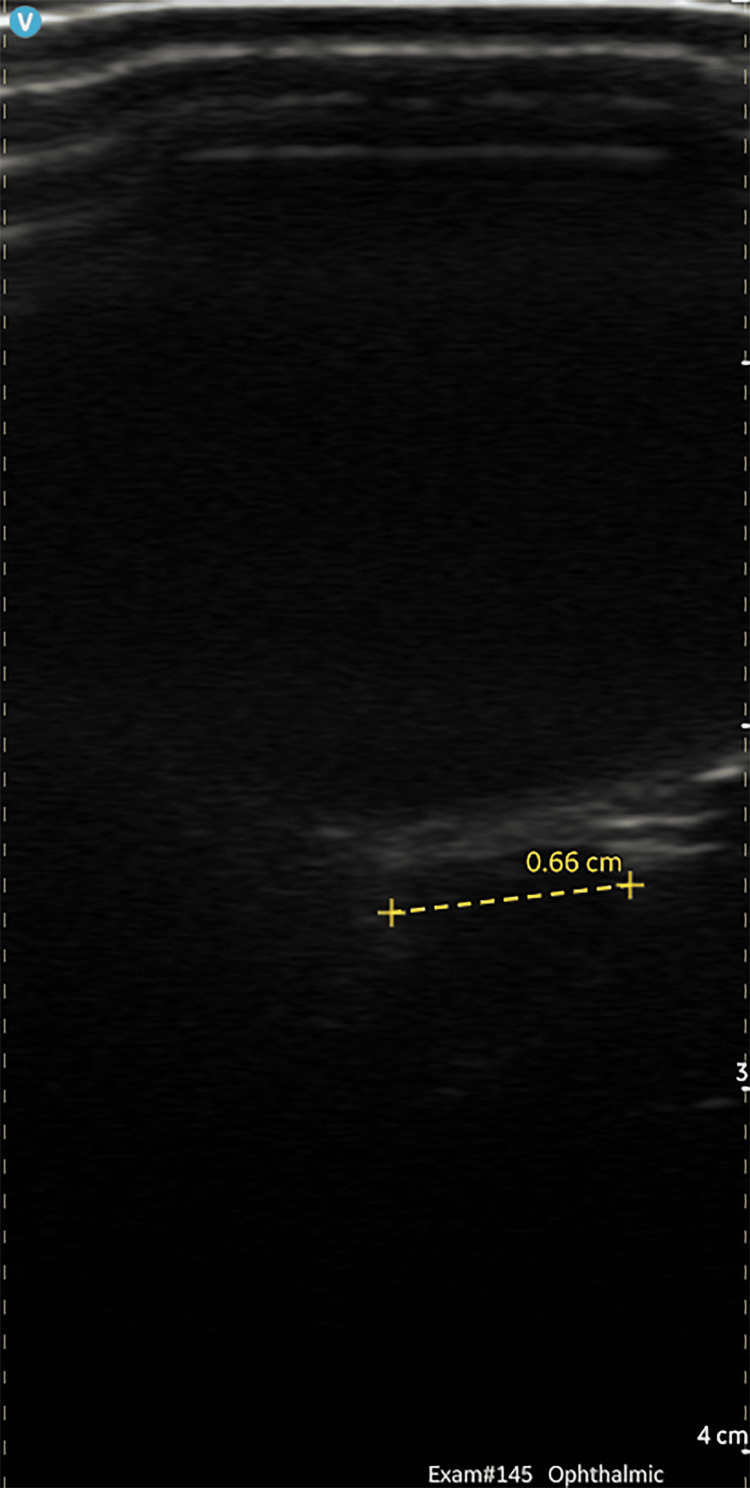
Ocular ultrasound of the right eye demonstrating an increased optic nerve sheath diameter (ONSD) measuring 6.6 mm. An ONSD greater than 5.0–5.7 mm is suggestive of raised intracranial pressure.

The patient presented with a postural headache, associated with pulsatile tinnitus and features suggestive of raised ICP. Bedside fundoscopy was inconclusive due to technical difficulty, and pupils were not dilated enough in that setting. Clinical examination was normal; however, ONSD ultrasonography demonstrated increased diameters of 6.6 mm (as shown in Figure 1) bilaterally, supporting a diagnosis of raised ICP.

In light of these findings, a non-contrast computed tomography (CT) scan of the head was performed to exclude an ICSOL, which returned unremarkable. OCT was subsequently arranged and showed no evidence of papilledema. The patient was discharged at that point as having no features of headache secondary to raised ICPs.

**Figure 2:**
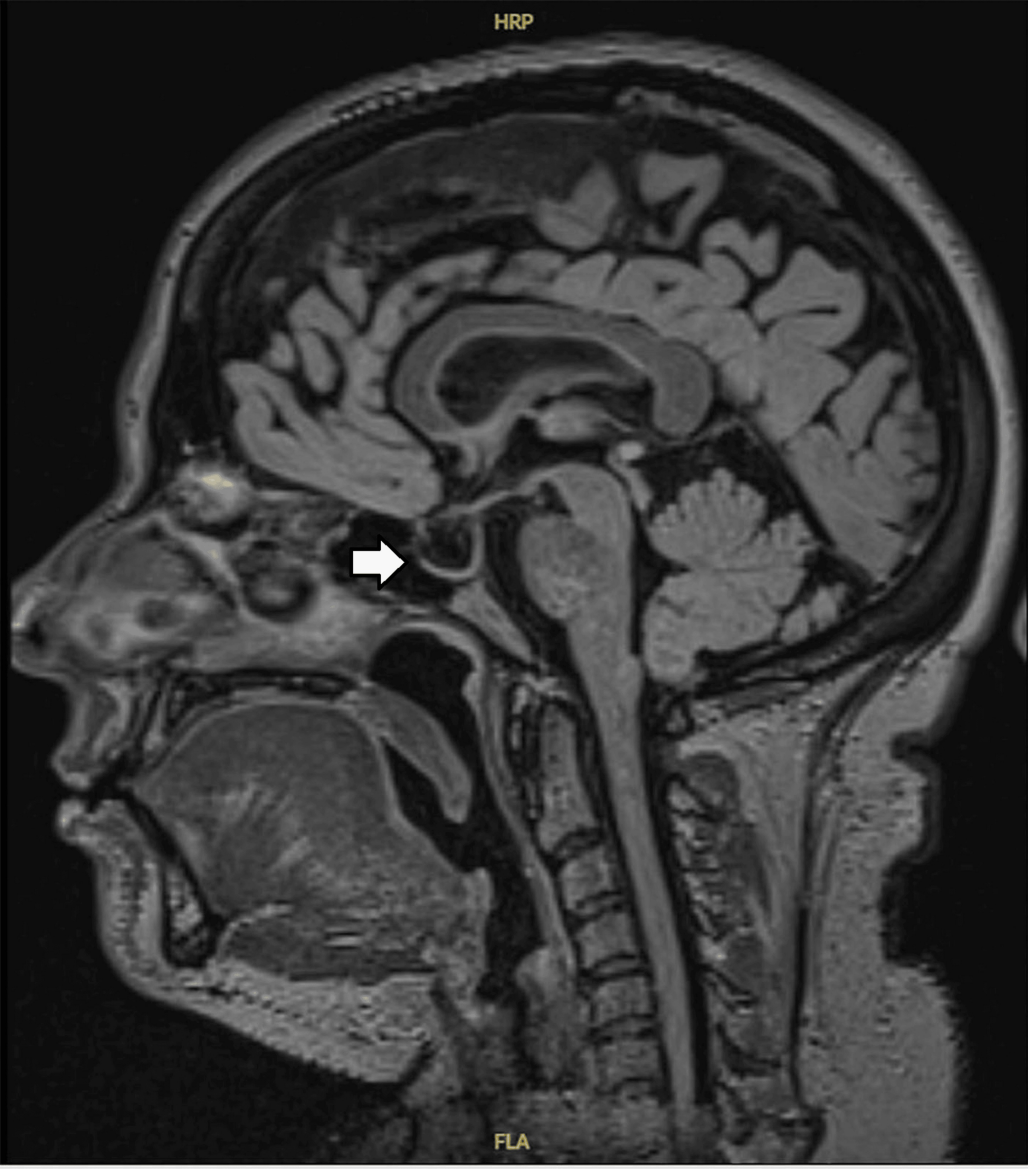
Sagittal T1-weighted MRI brain demonstrating features of empty sella, which appears partially filled with cerebrospinal fluid, resulting in a flattened pituitary gland against the floor of the sella-an imaging feature suggestive of idiopathic intracranial hypertension. MRI: magnetic resonance imaging

**Figure 3:**
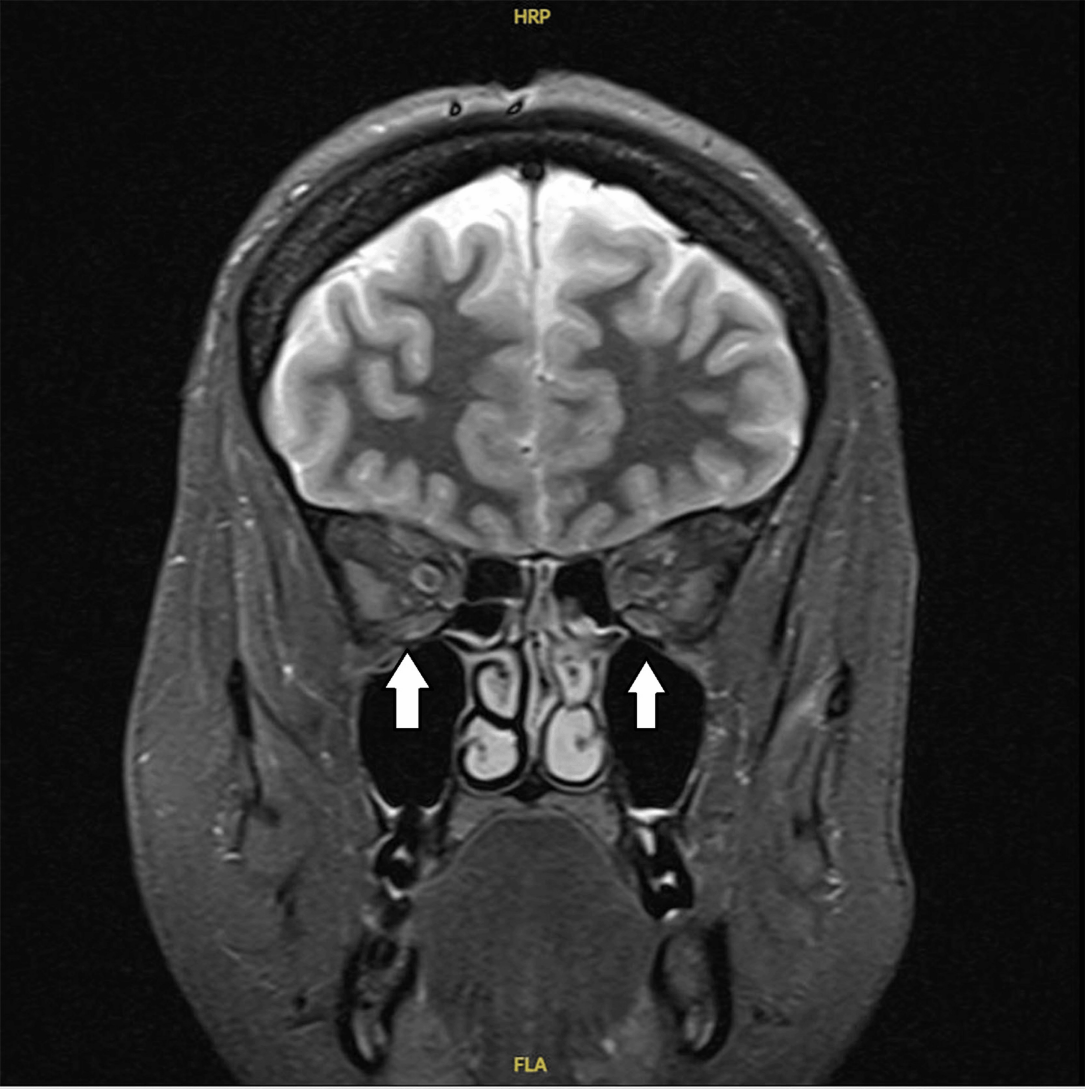
Coronal T2-weighted MRI image demonstrating bilateral optic nerve sheath prominence. The optic nerve sheaths appear distended, a radiological sign often associated with raised intracranial pressure. MRI: magnetic resonance imaging

**Figure 4:**
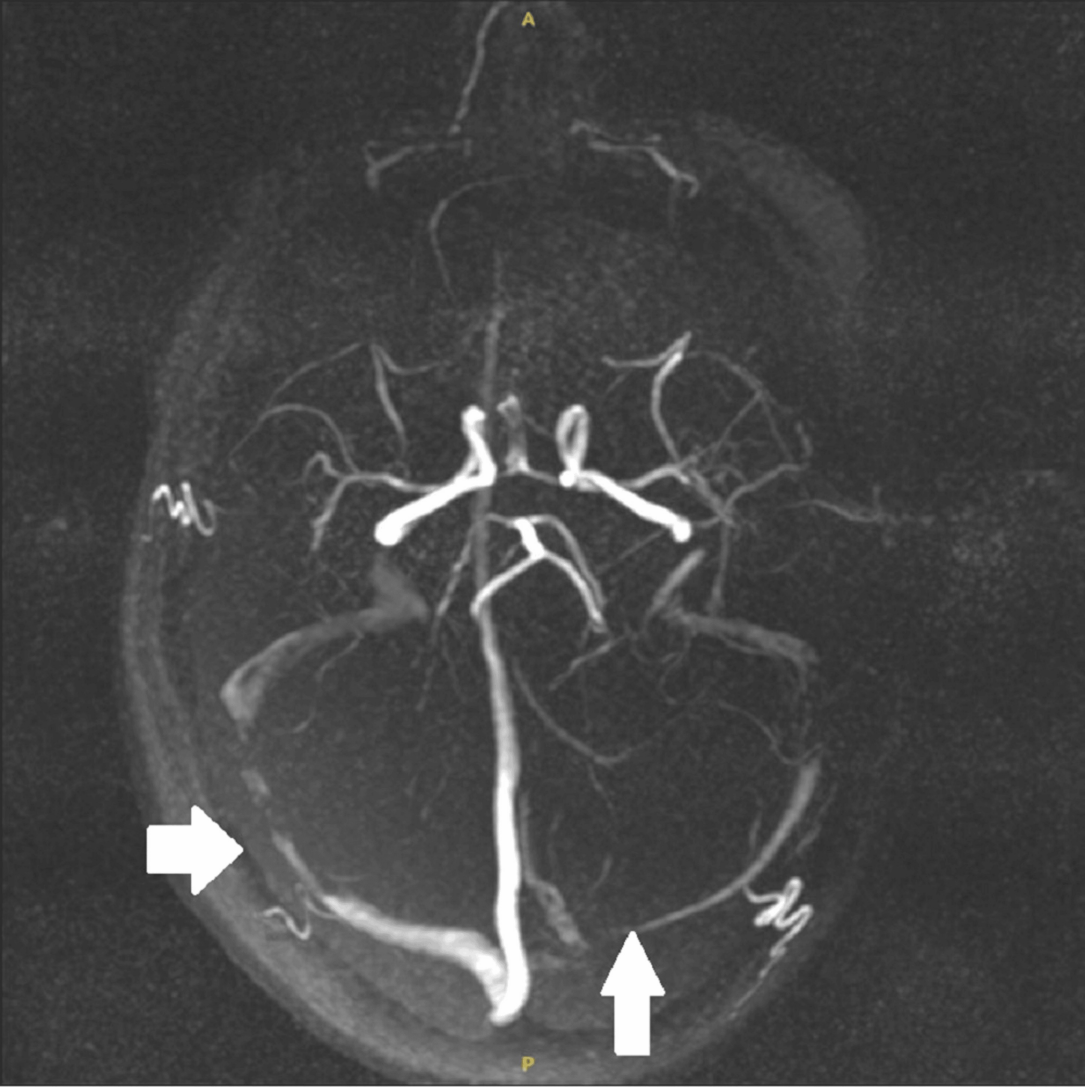
MRV showing stenosis (marked arrow) along the transverse sinuses, a finding often associated with idiopathic intracranial hypertension. MRV: magnetic resonance venography

Despite discharge, the patient continued to experience symptoms and was reviewed again in the clinic. A CT venogram was organized, which did not show any venous sinus thrombosis but raised suspicion of IIH showing narrowing of the transverse sinus. However, given the absence of any established features of raised ICP on OCT, a lumbar puncture (LP) was initially deferred, and neurologists instead advised an outpatient magnetic resonance imaging (MRI) brain and magnetic resonance venography (MRV) of the intracranial vessels. MRI and MRV brain revealed radiological features consistent with IIH-empty sella (Figure 2), bilateral optic nerve sheath distension (Figure 3), stenosis of the transverse sinuses (Figure 4), and mild flattening of the posterior sclera with no evidence of CVST.

Based on these findings, the neurology team ultimately proceeded with a LP, which confirmed raised opening pressure of 30 cm H_2_0, consistent with a diagnosis of IIH. The cerebrospinal fluid (CSF) profile was normal, including normal white cell count and normal protein and glucose. The patient was started on acetazolamide and has been planned to be followed up by the neurology outpatient clinic.

## Discussion

IIH is classically associated with papilledema, yet an important subset of patients-particularly adults-present with typical symptoms of raised ICP but no optic disk swelling. This diagnostic gray zone, known as IIH without papilledema (IIHWOP), remains under-recognized and is often misclassified as a primary headache disorder [2].

The absence of papilledema does not negate the possibility of IIH. Studies have consistently shown that between 5% and 14% of adult IIH patients do not exhibit optic disk edema at the time of presentation [3-5]. Despite this, their clinical symptoms-such as persistent positional headaches, pulsatile tinnitus, and transient visual disturbances-mirror those seen in classic IIH. The risk in these cases lies in the delay of diagnosis due to overreliance on fundoscopy and OCT, often leading to prolonged morbidity.

In the absence of papilledema, clinicians must turn to neuroimaging and non-invasive assessments to support their diagnostic reasoning. MRI findings such as an empty sella, flattened posterior globes, optic nerve sheath distension, and tortuosity of the optic nerves have all been associated with raised ICP [6,7]. Importantly, the revised diagnostic criteria proposed by Friedman et al. in 2013 allow for a diagnosis of IIHWOP if at least three of these four imaging features are present, in the context of elevated opening pressure and normal CSF composition [8].

Among these radiological features, transverse venous sinus stenosis has received particular attention. Often seen bilaterally, this finding is thought to either contribute to or result from impaired venous drainage in the context of elevated ICP. Farb et al. reported that the vast majority of patients with IIH demonstrate such stenoses, making it a valuable imaging biomarker even in patients without papilledema [9].

Another powerful tool in the diagnostic process is ONSD measurement via ocular ultrasound. The optic nerve sheath, as an extension of the subarachnoid space, becomes distended with elevated ICP. Several studies have demonstrated a strong correlation between ONSD and invasively measured ICP, with thresholds around 5-5.7 mm providing good sensitivity and specificity for raised pressure [10-12]. This makes ONSD ultrasonography especially valuable in cases where MRI is equivocal or where LP must be delayed.

What this case reinforces is the importance of a high index of suspicion. When an adult presents with classical IIH symptoms-particularly positional headache, pulsatile tinnitus, and visual obscurations-absence of papilledema on examination should not derail the investigation. Instead, it should prompt a thorough evaluation with MRI/MRV and, where available, ONSD ultrasonography. A diagnostic LP remains the gold standard, but by the time this is performed, the imaging findings and clinical features should already be raising the right questions.

Timely recognition of IIHWOP is crucial. Without it, patients may be labelled as having refractory migraine or functional symptoms, delaying effective treatment. Yet with appropriate intervention-including weight management, acetazolamide, and, where necessary, procedural interventions-the prognosis is often good [13,14]. The key is seeing beyond the fundus and recognizing that raised ICP can still be present in the quiet absence of papilledema.

## Conclusions

This case highlights the diagnostic complexity of IIH in the absence of papilledema, a feature traditionally considered central to its identification. As illustrated, patients may present with classical symptoms yet elude diagnosis through standard initial investigations. ONSD ultrasound, as a non-invasive and accessible modality, can provide critical early clues in such atypical presentations. Incorporating ONSD assessment into the diagnostic algorithm for suspected IIH may facilitate earlier recognition, reduce reliance on more invasive procedures, and ultimately improve patient outcomes.
